# A visualized nomogram to online predict futile recanalization after endovascular thrombectomy in basilar artery occlusion stroke

**DOI:** 10.3389/fneur.2022.968037

**Published:** 2022-08-26

**Authors:** ShiTeng Lin, XinPing Lin, Juan Zhang, Meng Wan, Chen Chen, Qiong Jie, YueZhang Wu, RunZe Qiu, XiaoLi Cui, ChunLian Jiang, JianJun Zou, ZhiHong Zhao

**Affiliations:** ^1^School of Basic Medicine and Clinical Pharmacy, China Pharmaceutical University, Nanjing, China; ^2^Department of Clinical Pharmacology, Nanjing First Hospital, Nanjing Medical University, Nanjing, China; ^3^Department of Neurology, Nanjing Yuhua Hospital, Yuhua Branch of Nanjing First Hospital, Nanjing Medical University, Nanjing, China; ^4^Department of Pharmacy, Nanjing First Hospital, China Pharmaceutical University, Nanjing, China; ^5^Department of Pathology, Nanjing First Hospital, Nanjing Medical University, Nanjing, China; ^6^Department of Neurology, The First Affiliated Hospital (People's Hospital of Hunan), Hunan Normal University, Changsha, China

**Keywords:** basilar artery occlusion, futile recanalization, endovascular thrombectomy, nomogram model, predictive model

## Abstract

**Background and purpose:**

Futile recanalization occurs in a significant proportion of patients with basilar artery occlusion (BAO) after endovascular thrombectomy (EVT). Therefore, our goal was to develop a visualized nomogram model to early identify patients with BAO who would be at high risk of futile recanalization, more importantly, to aid neurologists in selecting the most appropriate candidates for EVT.

**Methods:**

Patients with BAO with EVT and the Thrombolysis in Cerebral Infarction score of ≥2b were included in the National Advanced Stroke Center of Nanjing First Hospital (China) from October 2016 to June 2021. The exclusion criteria were lacking the 3-month Modified Rankin Scale (mRS), age <18 years, the premorbid mRS score >2, and unavailable baseline CT imaging. Potential predictors were selected for the construction of the nomogram model and the predictive and calibration capabilities of the model were assessed.

**Results:**

A total of 84 patients with BAO were finally enrolled in this study, and patients with futile recanalization accounted for 50.0% (42). The area under the curve (AUC) of the nomogram model was 0.866 (95% CI, 0.786–0.946). The mean squared error, an indicator of the calibration ability of our prediction model, was 0.025. A web-based nomogram model for broader and easier access by clinicians is available online at https://trend.shinyapps.io/DynNomapp/.

**Conclusion:**

We constructed a visualized nomogram model to accurately and online predict the risk of futile recanalization for patients with BAO, as well as assist in the selection of appropriate candidates for EVT.

## Introduction

Basilar artery occlusion (BAO) strokes represent only 1% of all the ischemic strokes, but are devastating for patients (1). The American Heart Association/American Stroke Association guidelines indicated that endovascular thrombectomy (EVT) should be considered reasonable for carefully selected patients with BAO stroke (2). The therapeutic goal of EVT is to achieve endovascular recanalization to improve long-term functional outcomes. However, a substantial proportion of patients experience successful reperfusion, but fail to achieve favorable outcomes, defined as “futile recanalization.” Recently, several studies of literature have reported that futile recanalization occurred in more than half of the BAO (3–5). Prior studies in patients suffering from large vessel occlusion with anterior circulation stroke have demonstrated that futile recanalization depends on patient-specific factors, as well as procedural considerations, such as age, the admission National Institutes of Health Stroke Scale (NIHSS), and the number of stent retriever passes (6–9). However, those factors remain elusive in BAO.

Nomogram is a reliable and visual statistical instrument that has the ability to develop a continuous scoring system by incorporating different data. By creating an intuitive graph, a nomogram derives the risk probability of a clinical event and divides the patients into two different groups. Although the ATTENTION trial demonstrated a significant effect among patients with BAO with the baseline NIHSS ≥ 10 undergoing EVT, the efficacy of stroke thrombectomy is largely determined by patient selection (10). Therefore, a nomogram model to predict futile recanalization that aimed to inform decision support in selecting patients with BAO for EVT is important. However, there are no reliable nomogram models developed with this target in mind.

Here, we aimed to identify the predictors of futile recanalization in patients with BAO and to develop a visualized nomogram aimed to assist clinicians in evaluating the risk of futile recanalization in this population, and more importantly, providing individualized information in selecting the most appropriate candidates for EVT.

## Materials and methods

### Study population

We retrospectively collected all the patients who received BAO in the National Advanced Stroke Center of Nanjing First Hospital (China) from October 2016 to June 2021. Patients were included if they underwent EVT and had the Thrombolysis in Cerebral Infarction (TICI) score of ≥ 2b. Patients were excluded from the study in the case of lack of the 3-month Modified Rankin Scale (mRS), age <18 years, the premorbid mRS score > 2, and unavailable baseline CT imaging.

The present research was approved by the Ethics Committee of Nanjing First Hospital (document number: KY20130424-01) and informed consent was obtained for each participant.

### Patient clinical and radiological variables

Related clinical and radiological variables were routinely recorded for individual patients. Demographic data included age, sex, body mass index (BMI), and years of education. Risk factors of vessels included hypertension, diabetes, dyslipidemia, coronary artery disease, and previous stroke history. Laboratory data included fasting blood glucose (FBG), systolic blood pressure (SBP), diastolic blood pressure (DBP), platelet count, and lipid testing indicators. Ischemic stroke etiology was classified by the Trial of ORG 10172 in Acute Stroke Treatment (TOAST) criteria (11).

The extent of early infarct was measured by the posterior circulation Acute Stroke Prognosis Early CT Score (pc-ASPECTS) with CT or MRI, which was assessed by two independent neurologists. Measurements from the diagnostic modality provided scores ranging from 0 to 10, with the higher scores representing smaller early ischemic changes.

We recorded blood pressure data at 1, 3, 6, 12, and 24 h after EVT. Then, the SD and coefficient of variation (CV) of systolic and diastolic blood pressures were calculated using data from these five time points. In addition, we have collected the following five points: onset to emergency (OTE), onset to image (OTI), onset to puncture (OTP), onset to recanalization (OTR), and puncture to recanalization (PTR).

### Patient outcome

We divided eligible patients with the mTICI ≥ 2b into two groups using the 90-day mRS score, which included futile recanalization (the 90-day mRS of 3–6) and meaningful recanalization groups (the 90-day mRS of 0–2). The 90-day mRS was collected by telephone interview or outpatient 3 months 90 days after onset.

### Statistical analysis

The Shapiro–Wilk test was carried out to test the normality of continuous variables. Normally distributed continuous variables were presented by their mean and SD, while non-normally distributed continuous variables were presented by their median and interquartile range (IQR). The Mann–Whitney *U*-test and *t*-test were used for the comparison of normally distributed continuous variables with non-normally distributed continuous variables, respectively. Categorical data were tested using the Pearson's chi-squared test or Fisher's exact test, expressed as the percentages of events. Factors with more than 10% missing data were excluded and mean imputation was used with low missing data. All the tests were two-sided and *p*-values <0.05 were considered to be statistically significant.

### Development and assessment of the models

Variables with a value of *p* < 0.05 in the univariate analysis were re-entered into the multivariate logistic regression model in a backward stepwise method. Also, the odds ratio and 95% CI were presented for potential predictors incorporated in the multivariate logistic regression models. Finally, the selected potential predictors were used to construct the nomogram model. Each variable in the nomogram was given a weighted score, which was then summed to create a total score and finally converted to individual risk of futile recanalization by the function between the total score and the probability of the outcome. The “rms” package with R software was used to build a conventional nomogram model.

The area under the receiver operating characteristic curves (AUCs) were used to assess the model's predictive ability and to determine the thresholds that separate the meaningful recanalization and futile recanalization groups. The Youden index (sensitivity + specificity −1) was calculated for different vs. thresholds, and the score at the greatest Youden index was used as the cutoff value. Based on the greatest Youden index, sensitivity and specificity were calculated. We assessed calibration through calibration plots and mean absolute error. A calibration plot was generated with 1,000 bootstrap resampling for depicting the correlation between the actual unfavorable outcome and the predicted probability of an unfavorable outcome. The “DynNom” package with R software was used to build dynamic nomogram models for the prediction of an unfavorable outcome in patients with BAO at 3 months on the Internet (12). The above data analysis was implemented with SPSS version 25.0 (IBM Corporation, Armonk, New York, USA) and R statistical software version 4.1.0.

## Results

### Study population

**Figure 1:**
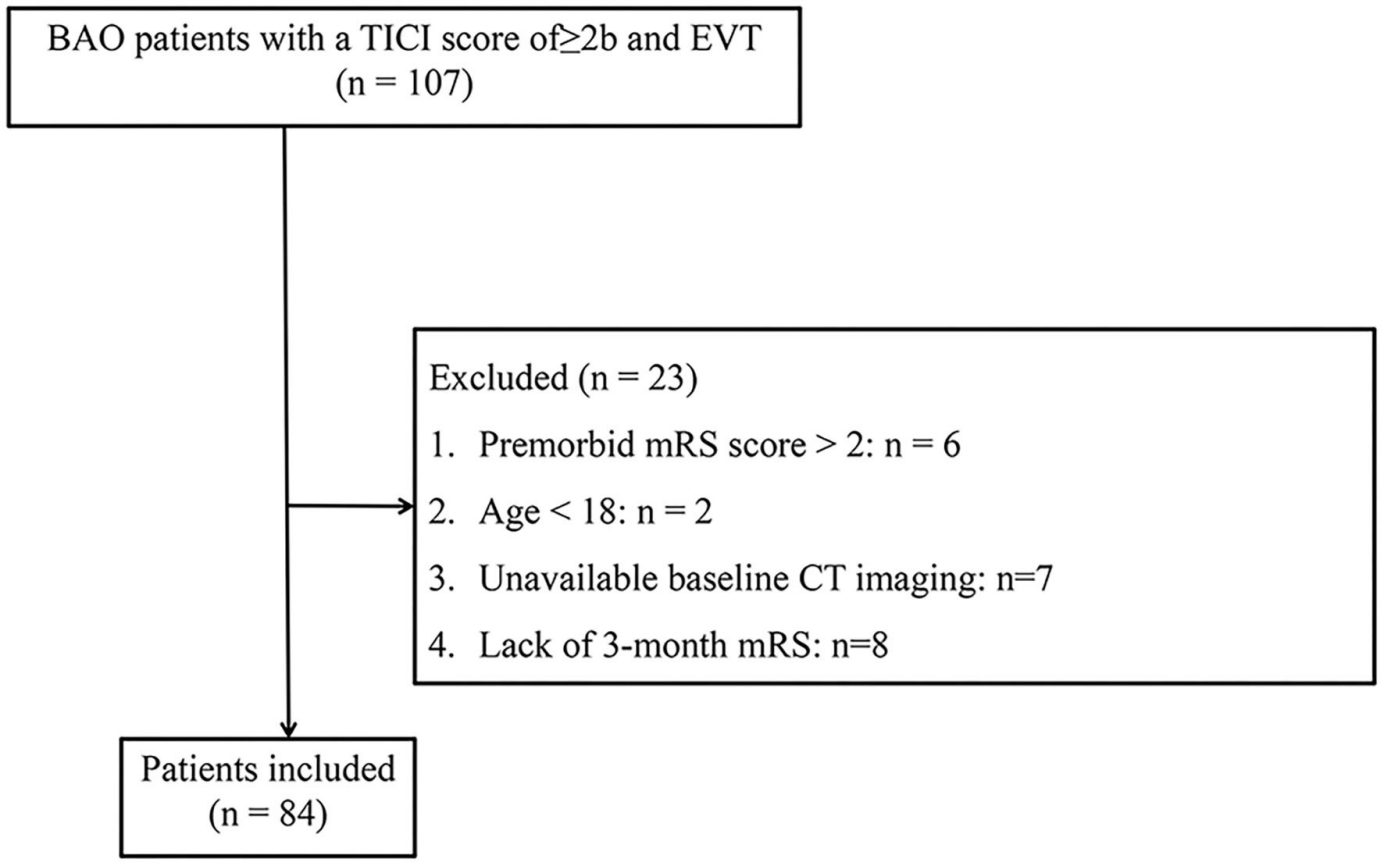
Flowchart of patient inclusion and exclusion criteria.

Of the 107 patients with BAO who were admitted to our institution and underwent EVT first registered, 23 patients were not included in the study population. The specific process of exclusion for 23 patients was shown in Figure 1 and a total of 84 patients were eventually included in the study population. Futile recanalization was observed in 42 (50.0%) patients at 3 months.

The clinical and radiological characteristics of the whole cohort (*n* = 84), the meaningful recanalization cohorts (*n* = 42), and futile recanalization (*n* = 42) cohorts are given in Table 1A. The median age of the study population included was 65.87 (SD = 12.04) years and 68 (81.0%) patients were men. The median NIHSS score on admission, the pc-ASPECTS, and the premorbid mRS were 16.00 (IQR, 8.00–29.75), 8.5 (IQR, 7.0–10.0), and 0 (IQR, 0–0), respectively. A history of the previous stroke was observed in 21 of the 84 patients (25.0%).

**Table 1A:** Demographics and clinical characteristics.

	**Total**	**Meaningful recanalization**	**Futile recanalization**	***p*-value**
	**(*n* = 84)**	**(*n* = 42)**	**(*n* = 42)**	
**Baseline characteristics**
Age, years, mean (SD)	65.87 (12.04)	64.40 (13.10)	67.33 (10.84)	0.268
Male sex, *n* (%)	68 (81.0)	32 (76.2)	36 (85.7)	0.266
BMI, kg/m^2^, mean (SD)	24.67 (3.49)	24.48 (3.74)	24.85 (3.26)	0.631
Education, years, *n* (%)				0.863
0–6	38 (45.2)	19 (45.2)	19 (45.2)	
6–9	17 (20.2)	11 (26.2)	6 (14.3)	
9–12	18 (21.4)	7 (16.7)	11 (26.2)	
>12	11 (13.1)	5 (11.9)	6 (14.3)	
Premorbid mRS (IQR)	0 (0–0)	0 (0–0)	0 (0–0)	0.081
NIHSS on admission, median (IQR)	16.00 (8.00–29.75)	11.50 (5.00–18.25)	25.00 (16.00–35.00)	<0.001
Baseline SBP, mmHg, mean (SD)	140.89 (24.42)	142.93 (23.87)	138.86 (25.08)	0.448
Baseline DBP, mmHg, mean (SD)	82.67 (15.35)	82.43 (15.69)	82.90 (15.18)	0.888
Platelet count, 109/L, mean (SD)	200.19 (55.089)	204.52 (58.94)	195.86 (51.30)	0.474
FBG, mmol/L, median (IQR)	6.62 (5.60–8.29)	6.63 (5.46–7.73)	6.62 (5.76–8.67)	0.408
TC, mmol/L, median (IQR)	4.73 (3.90–5.42)	4.74 (4.05–5.33)	4.71 (3.74–5.85)	0.865
TG, mmol/L, median (IQR)	1.17 (0.81–1.85)	1.19 (0.84–1.76)	1.05 (0.79–1.97)	0.668
HDL, mmol/L, median (IQR)	1.07 (0.86–1.25)	1.11 (0.83–1.29)	1.06 (0.86–1.20)	0.806
LDL, mmol/L, median (IQR)	3.05 (2.43–3.44)	3.09 (2.54–3.44)	2.85 (2.28–3.44)	0.393
**Risk factors of vessels**
Hypertension, *n* (%)	65 (77.4)	32 (76.2)	33 (78.6)	0.794
Diabetes mellitus, *n* (%)	27 (32.1)	14 (33.3)	13 (31.0)	0.815
Dyslipidemia, *n* (%)	35 (41.7)	17 (40.5)	18 (42.9)	0.825
Coronary artery disease, *n* (%)	11 (13.1)	6 (14.3)	5 (11.9)	0.746
Atrial fibrillation, *n* (%)	20 (23.8)	10 (23.8)	10 (23.8)	1.000
Previous stroke, *n* (%)	21 (25.0)	5 (11.9)	16 (38.1)	0.006
Smoking, *n* (%)				0.952
Never smoker	34 (40.5)	18 (42.9)	16 (38.1)	
Former smoker	10 (11.9)	5 (11.9)	5 (11.9)	
Current smoker	40 (47.6)	19 (45.2)	21 (50.0)	
Drinking, *n* (%)				0.239
Never drinker	52 (61.9)	24 (57.1)	28 (66.7)	
Former drinker	3 (3.6)	3 (7.1)	0 (0.0)	
Current drinker	29 (34.5)	15 (35.7)	14 (33.3)	
**Radiological baseline characteristics**
Pc-ASPECTS on admission, median (IQR)	8.5 (7.0–10.0)	9.0 (8.0–10.0)	7.5 (6.0–9.0)	<0.001
**Cause of stroke**, ***n*** **(%)**
LAA	61 (72.6)	32 (76.2)	29 (69.0)	0.463
CE	16 (19.0)	5 (11.9)	11 (26.2)	0.095
SAO	1 (1.2)	1 (2.4)	0 (0.0)	1.000
SOC	3 (3.6)	2 (4.8)	1 (2.4)	1.000
SUC	3 (3.6)	2 (4.8)	1 (2.4)	1.000
**Vascular occlusion site**, ***n*** **(%)**
Vertebral artery	36 (42.9)	15 (35.7)	21 (50.0)	0.186
Basilar artery	48 (57.1)	27 (64.3)	21 (50.0)	0.186
**Medication use history**
Previous antiplatelet, *n* (%)	7 (8.3)	4 (9.5)	3 (7.1)	1.000
Previous anticoagulation, *n* (%)	5 (6.0)	3 (7.1)	2 (4.8)	1.000
Previous statin, *n* (%)	5 (6.0)	1 (2.4)	4 (9.5)	0.360

IQR, interquartile range; SD, standard deviation; BMI, body mass index; mRS, modified Ranking Scale; NIHSS, National Institutes of Health Stroke Scale; SBP, systolic blood pressure; DBP, diastolic blood pressure; Pc-ASPECTS, posterior circulation Acute Stroke Prognosis Early CT Score; LAA, large artery atherosclerosis; CE, cardioembolism; SAO, small artery occlusion; SOC, stroke of other determined cause; SUC, stroke of undetermined cause.

Patients' treatment information and complication are shown in Table 1B. In the whole cohort, thirty-two patients (38.1%) received intravenous thrombolysis. Twenty-two of the 84 patients (26.2%) achieved the TICI score of 2b and the remaining 62 patients (73.8%) achieved the TICI score of 3. The median number of passages in the study population was 1 (IQR, 1–2) and symptomatic intracranial hemorrhage (sICH) was observed in 6 (7.1%) patients from the entire cohort.

**Table 1B:** Treatment information and complications.

	**Total**	**Meaningful recanalization**	**Futile**	***p*-value**
	**(*n* = 84)**	**(*n* = 42)**	**(*n* = 42)**	
**Treatment information**
Intravenous thrombolysis, *n* (%)	32 (38.1)	18 (42.9)	14 (33.3)	0.369
Number of passages, *n* (%)	1 (1–2)	1 (1–2)	1 (1–2)	0.444
Onset to emergency, min, median (IQR)	180.00 (105.00–325.00)	207.50 (83.75–336.25)	177.50 (112.50–316.25)	0.816
Onset to image, min, median (IQR)	271.00 (160.25–380.00)	287.50 (157.50–407.25)	228.00 (163.25–369.25)	0.447
Onset to groin, min, median (IQR)	328.00 (230.00–458.75)	335.00 (229.00–535.75)	281.50 (230.00–413.50)	0.310
Onset to recanalization, min, median (IQR)	402.50 (309.75–542.75)	424.00 (330.25–613.25)	381.00 (297.50–520.25)	0.398
Groin to recanalization, min, median (IQR)	78.00 (57.75–105.75)	76.00 (60.00–102.75)	80.00 (55.00–115.00)	0.458
mTICI score, *n* (%)				0.620
2b	22 (26.2)	12 (28.6)	10 (23.8)	
3	62 (73.8)	30 (71.4)	32 (76.2)	
**Post-treatment blood pressure variability**
**SBP**
SD, median (IQR)	13.12 (8.59–18.54)	11.62 (7.60–19.01)	13.89 (9.92–17.52)	0.269
CV, median (IQR)	9.87 (6.76–14.03)	9.00 (6.14–15.44)	10.70 (7.55–12.66)	0.262
**DBP**
SD, median (IQR)	7.55 (5.19–11.60)	6.86 (5.01–10.07)	8.52 (5.50–12.98)	0.222
CV, median (IQR)	10.35 (6.49–16.13)	9.57 (6.16–12.46)	11.83 (6.79–16.84)	0.222
**Complications**
sICH, *n* (%)	6 (7.1)	0 (0)	6 (14.3)	0.026
Death in hospital	9 (10.7)	0 (0)	9 (21.4)	0.002
Respiratory infections, *n* (%)	59 (70.2)	28 (47.5)	31 (73.8)	0.474
Secondary epilepsy, *n* (%)	1 (1.2)	0 (0)	1 (2.4)	1.000
Gastrointestinal bleeding, *n* (%)	3 (3.6)	0 (0)	3 (7.1)	0.241

IQR, interquartile range; mTICI, modified Thrombolysis in Cerebral Infarction; SBP, systolic blood pressure; DBP, diastolic blood pressure; SD, standard deviation; CV, coefficient of variation; sICH, symptomatic intracranial hemorrhage.

### Univariate and multivariate analyses

In the univariate logistic analysis, the NIHSS on admission (*p* < 0.001), previous stroke (*P* = 0.006), and the pc-ASPECTS (*p* < 0.001) were found to be significantly associated with futile recanalization (Tables 1A,B).

The multivariate logistic regression analysis identified previous stroke (OR, 4.421; 95% CI, 1.112–17.587), the NIHSS on admission (OR, 1.111; 95% CI, 1.051–1.174), and the pc-ASPECTS (OR, 0.519; 95% CI, 0.352–0.767) as prognostic factors of an unfavorable outcome at 3 months (Table 2). The logistic regression model resulted: Log [*p*(x)/1–*p*(x)] = 3.1062 + (1.4865 × Previous stroke) + (0.1052 × NIHSS on admission) + (−0.6549 × pc-ASPECTS); where *p*(x) was the probability of futile recanalization. Patients with previous stroke, the higher NIHSS on admission, and the lower pc-ASPECTS were more likely to experience futile recanalization.

**Table 2:** The multivariate logistic regression analysis.

**Variable**	**OR**	**95% CI**	***P***
Previous stroke	4.42	1.11–17.59	0.035
NIHSS on admission	1.11	1.05–1.17	<0.001
Pc_ASPECTS	0.52	0.35–0.77	0.001

### Development and assessment of nomogram model

**Figure 2:**
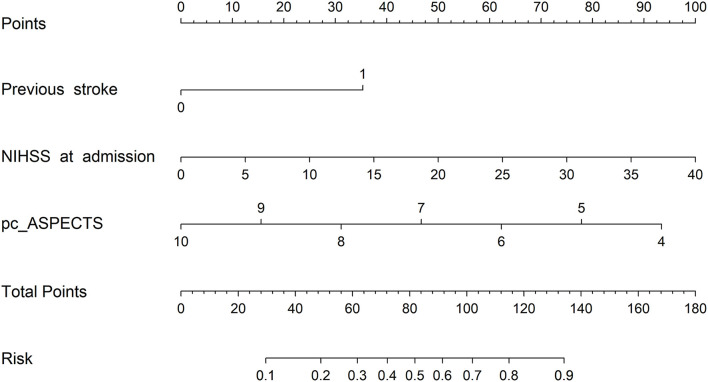
The prognostic nomogram of futile recanalization. Pc-ASPECTS, posterior circulation Acute Stroke Prognosis Early CT Score.

A prognostic nomogram was established for futile recanalization before EVT by integrating independent significant risk factors based on the multivariate logistic regression, which is shown in Figure 2. In addition, we established a dynamic web-based nomogram for broader and easier access by clinicians and researchers, which is available online at https://trend.shinyapps.io/DynNomapp/. Clinicians and researchers can input the individual variables of patients on the web page to obtain the risk of futile recanalization effortlessly.

**Figure 3:**
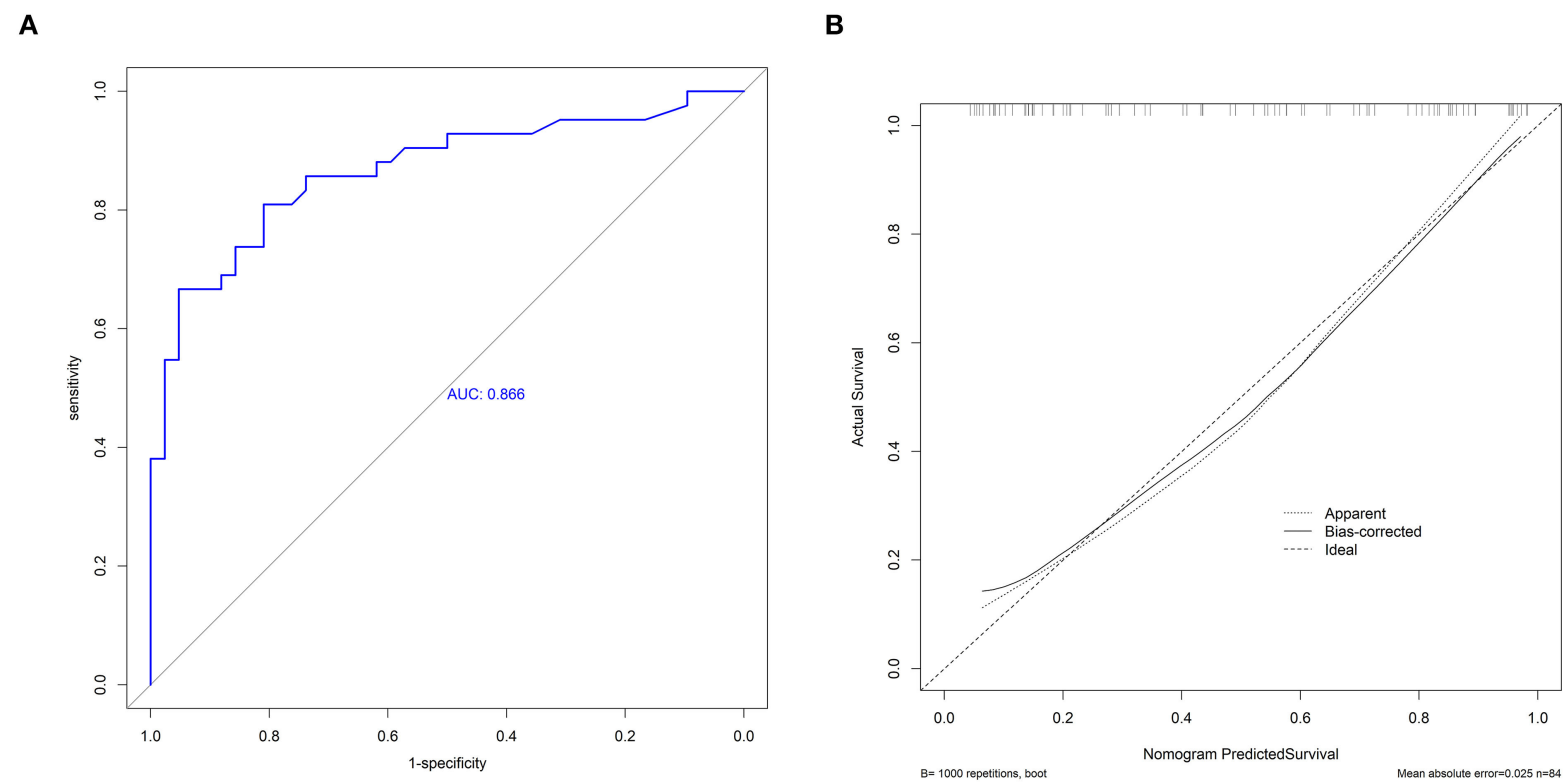
The receiver operating characteristic curve (ROC) of the nomogram **(A)** and the calibration plot of the nomogram **(B)**.

As shown in Figure 3A, the predictive performance was observed in the prognostic nomogram (AUC, 0.866; 95% CI, 0.786–0.946), which demonstrated the superior discriminatory ability of our model. The maximum Youden index was 0.507 with 81% sensitivity and 81% specificity. In addition, the points of the calibration plot for the probability of futile recanalization for patients with BAO are close to the 45° line, suggesting a positive correlation between predictions by nomogram and actual observations (Figure 3B). The mean squared error of the prognostic model was 0.025, also showing a strong level of calibration performance of the nomogram model we built.

## Discussion

In the present study, we developed a visualized nomogram for the evaluation of futile recanalization in patients with BAO. We found that previous stroke, the NIHSS on admission, and the pc-ASPECTS are potential predictors of futile recanalization *via* EVT in patients with BAO. Therefore, our nomogram model to quantify the risk of futile recanalization could aid in identifying risk factors, as well as a prediction for futile recanalization after EVT.

**Figure 4:**
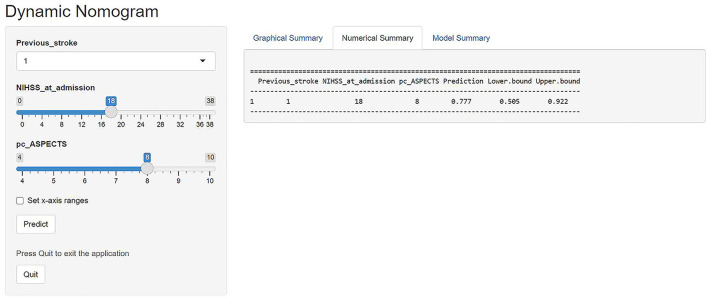
The example diagram of the visualized nomogram model.

A major strength of our study is that our prediction model demonstrated favorable predictive (AUC, 0.866; 95% CI, 0.786–0.946) and calibration capabilities (mean squared error = 0.025). Besides, in a previous study of a scoring scale for the prediction of futile recanalization of the posterior circulation (13), its predictors included the pons-midbrain index and bilateral thalamic infarction on diffusion-weighted imaging. These are difficult variables to obtain and are not widely used in clinical practice, especially in primary care, which limits the clinical dissemination of this scoring scale. In contrast, the three variables of our model are easily accessible and the visualized model is readily available for use. In addition, we provided an example to facilitate clinicians and researchers to better understand the utility of our web-based nomogram. If a patient had a history of stroke, the initial NIHSS score of 18, and the pc-ASPECTS of 8, as shown in Figure 4, the web page would calculate a 3-month nullification risk recanalization of 0.777 (95% CI, 0.505–0.922), which considered as a high-risk patient because the predicted probability is greater than the threshold of our model (0.507). When the “Graphical Summary” button was clicked, the site would display a graph of the predicted probabilities and their 95% CIs, and as we click on the “Model Summary” button, the site would provide information on the specific parameters of our model.

Although the baseline variables we included in the study at the outset were those of demographics, clinical characteristics, and treatment information, it so happened that all the variables that were eventually included in the model were preoperative. Thus, our model can be used preoperatively as a clinical decision support tool to assist physicians in deciding whether to perform EVT. Specifically, a patient may have undergone a successful thrombectomy with no improvement in prognosis, which results in a waste of medical resources and money. In addition, ineffective treatment will increase pain and discomfort at the end of patients' life, reducing the quality of patient survival and delaying palliative care. Therefore, futile recanalization does not confer actual benefit to the patient. In patients predicted by our model to be at high risk of futile recanalization, physicians may need to consider the need for EVT in the context of the individual patient's situation. For primary care hospitals, in particular, consideration should be given to whether patients should be advised to be transferred to undergo EVT. Furthermore, in addition to constructing a traditional nomogram model, the dynamic nomogram was also developed on a web page to facilitate accessibility for clinicians. Certainly, we need to emphasize that our nomogram model is only part of the decision system for EVT in patients with BAO. Our model can only assist the physician, who has to decide on the specific treatment modality, taking into account the individual patient's situation and his own experience.

In the present study, the rate of “futile recanalization” after EVT was up to 50% (42 out of 84 patients), which was similar to previous studies of successful recanalization in patients with BAO (13, 14). Several predictors of futile recanalization in patients with BAO undergoing EVT have been established in our study. The multivariate logistic regression analyses demonstrated that previous stroke was associated with a higher likelihood of futile recanalization, supposedly due to their greater age (15) and preexisting disabilities (16). Therefore, the overall potential for neurological rehabilitation was low. The present study indicates that a previous stroke was a predictor of poor clinical outcomes despite successful reperfusion, namely, was associated with the effectiveness of EVT. Although systemic thrombolysis was not effective in patients with the previous medium to large stroke, data on the effectiveness of EVT in patients with BAO with a previous stroke are lacking (17). EVT is a safe and effective procedure for patients with BAO with the previous stroke that remains to be better studied in future studies.

Generally, age and the NIHSS were recognized to be a predictor of prognosis in BAO stroke (3, 18). As shown in Table 2, the higher NIHSS on admission was significantly associated with a higher rate of futile recanalization in the present study. Such results are easy to understand because the NIHSS is a standardized stroke scale to quantify the degree of neurological deficit. The potential for neurological rehabilitation of the elderly was comparably lower compared with younger persons because of the preexisting cognitive and/or physical disabilities, and a higher rate of serious complications during hospitalization (19–21). It is of surprise that age was not independently associated with futile recanalization. This might be due to the differences in patient characteristics and a selection bias during treatment decisions. Although our findings challenge the data of the Endovascular Stroke Treatment (ENDOSTROKE) registry (3), they are in line with the results of Son et al. (14). It should be pointed out that age is a factor that physicians need to consider carefully when making decisions.

Our analysis also shows that the pc-ASPECTS was an independent predictor of futile recanalization. The pc-ASPECTS, first proposed by Puetz et al. (22), has been validated for grading irreversible ischemic in the posterior circulation and is often used to select patients with BAO who would most likely benefit from EVT, thus helping to improve clinical prognosis in patients with BAO. Several studies have reported that the pc-ASPECT score ≥ 8 on the initial image increases the benefit of EVT (23–25). Nevertheless, there are different views on the treatment threshold. A recent study conducted by Sang et al. (26) provided evidence for the efficiency of EVT with the pc-ASPECTS ≥ 5. The present study bypasses the use of the statistical expedient of the artificial cutoff at the non-categorical by introducing a linear equation to calculate the coefficient of the ASPECTS score and drafting a concise chart to aid decision-making for EVT.

There are several limitations to our study. First, although our study collected a certain number of variables at different points in time, some possible risk factors are missing associated with adverse outcomes in patients with BAO, such as posterior circulation collateral status and the pons-midbrain index. However, these variables are not widely used in the clinical setting and are not readily available. Second, patients with BAO were all collected from the same hospital; therefore, our model suffered from a lack of external validation and the generalizability performance of the model still needs to be tested on patients from other institutions. In addition, as with other retrospective studies, our study has the drawback of selection bias such as patient exclusion due to missing data. So, we provided as much detail as possible about the patient's baseline information to facilitate further use or comparison of our model by other institutions or researchers. Third, the dichotomy of the mRS scores ignores the differences between the mRS scores 3, 4, 5, and 6; thus, this approach may not reflect subtle differences in functional outcomes of patients.

## Conclusion

This study demonstrated that the construction of our dynamic and visualized nomogram model could be applied preoperatively and online to accurately predict the risk of futile recanalization in patients with BAO and, thus, assist in the selection of clinical treatment modalities. In the future, subsequent multicenter studies will be more beneficial to the utility of our model in the clinical setting.

## Data availability statement

The raw data supporting the conclusions of this article will be made available by the authors, without undue reservation.

## Ethics statement

The studies involving human participants were reviewed and approved by the Ethics Committee of Nanjing First Hospital (document number: KY20130424-01). The patients/participants provided their written informed consent to participate in this study.

## Author contributions

SL: statistical analyses and drafting the manuscript. XL: drafting the manuscript. JZh: manuscript revision. MW, CC, QJ, YW, and RQ: data collection. XC, CJ, and ZZ: scientific supervision. JZo: manuscript revisions, handled funding, and supervision.

## Funding

This study was supported by the National Natural Science Foundation of China (82173899), the Jiangsu Pharmaceutical Association (H202108 and A2021024), the Hunan Natural Science Foundation (2021JJ70021), and the Hunan innovation guidance grant of clinical medical technology(2020SK50920).

## Conflict of interest

The authors declare that the research was conducted in the absence of any commercial or financial relationships that could be construed as a potential conflict of interest.

## Publisher's note

All claims expressed in this article are solely those of the authors and do not necessarily represent those of their affiliated organizations, or those of the publisher, the editors and the reviewers. Any product that may be evaluated in this article, or claim that may be made by its manufacturer, is not guaranteed or endorsed by the publisher.
